# Atypical cutaneous presentation of tuberous sclerosis complex: Giant angiofibroma on the scalp

**DOI:** 10.1590/0004-282X-ANP-2021-0315

**Published:** 2021-12-17

**Authors:** Leonardo Furtado Freitas, Leomar Benicio Maia Segundo, Danilo Manuel Cerqueira Costa, Márcio Luís Duarte, Luís Antônio Tobaru Tibana

**Affiliations:** 1 Universidade Federal de São Paulo, Departamento de Radiologia, São Paulo SP, Brazil.; 2 Universidade Federal de São Paulo, Departamento de Saúde Baseada em Evidências, São Paulo SP, Brazil.

Tuberous sclerosis (TSC) is an autosomal dominant neurocutaneous syndrome characterized by several abnormalities, including benign tumors of the embryonic ectoderm in multiple organs, such as skin, eyes, and central nervous system^1^. The main dermatological manifestations of TSC are hypochromic macules (ash leaf spots), facial angiofibromas, fibrous cephalic plaques, periungual fibroids, shagreen patch, and confetti lesions^2^.

A 26-year-old woman presented with a giant angiofibroma with an atypical and rare symptom of TSC, the main symptom being the skin lesions (Figures 1, 2, 3 and 4). The giant and asymmetric form is described as a rare presentation in the literature^3^.

**Figure 1:**
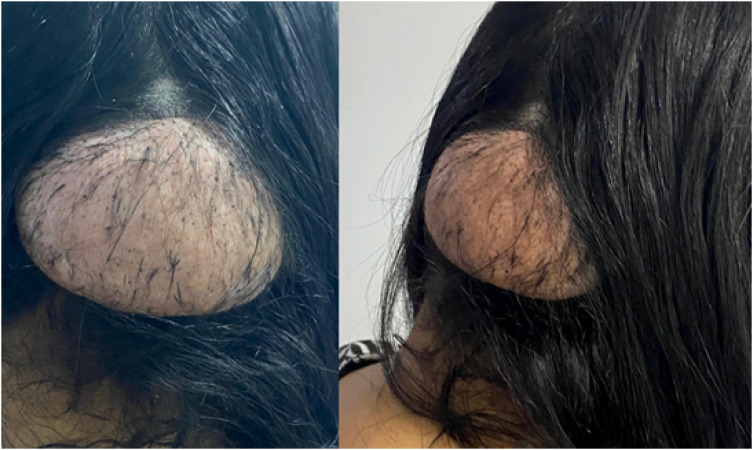
Massive lesion of soft parts in the occipital region presenting fibroelastic consistency, compatible with giant angiofibroma.

**Figure 2:**
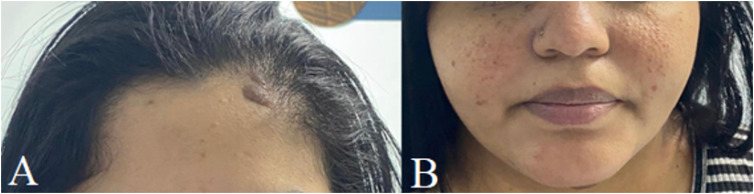
Hyperchromic papule on the left forehead (A) and small hyperchromic papular lesions in the malar regions (B).

**Figure 3:**
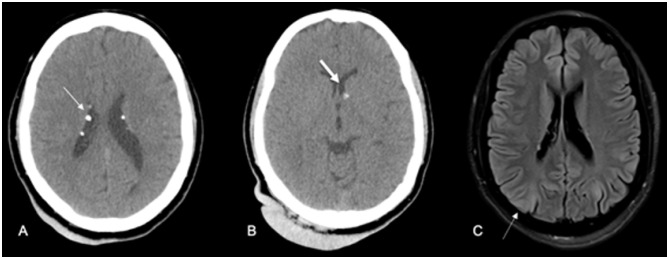
Typical lesions of tuberous sclerosis. In CT scans (A and B), it is possible to recognize subependymal nodules, some of which are calcified (arrow in A) and found in the topography of the left Monro foramen (arrow in B). Also, note the presence of giant occipital angiofibroma in these CT scans (A and B). FLAIR-weighted MRI image (C) showing evidence of hypersignal in the white and gray matters compatible with tubers.

**Figure 4:**
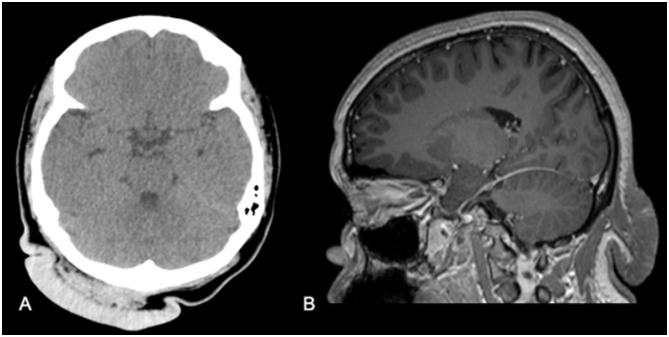
CT (A) and MRI scans (B) showing a soft tissue lesion characterized by marked cutaneous thickening in the occipital region.

## References

[1] Umeoka S, Koyama T, Miki Y, Akai M, Tsutsui K, Togashi K (2008). Pictorial review of tuberous sclerosis in various organs. Radiographics.

[2] Portocarrero LKL, Quental KN, Samorano LP, Oliveira ZNP, Rivitti-Machado MCDM (2018). Tuberous sclerosis complex: review based on new diagnostic criteria. An Bras Dermatol.

[3] Samia Y, Yousra C, Monia Y, Narjes M, Mouna A, Amel B (2014). Giant Angiofibroma associated with tuberous sclerosis: a case report. Research.

